# Experimental and Numerical Investigations on Compressive Performance of Additively Manufactured PLA Structures with Various Infill Patterns

**DOI:** 10.3390/polym18151845

**Published:** 2026-07-28

**Authors:** Alexandra Llidó Barragán, Aritz Unamuno Garay, Santiago Ferrándiz-Bou, Dana Luca Motoc, Cristina Pavón

**Affiliations:** 1 Instituto Universitario de Investigación de Tecnología de Materiales, Universitat Politècnica de València, Plaza Ferrándiz y Carbonell, 03801 Alcoy, Spain; aunagar@epsa.upv.es (A.U.G.); sferrand@mcm.upv.es (S.F.-B.); cripava1@epsa.upv.es (C.P.); 2 Department of Automotive and Transport Engineering, Transilvania University of Brasov (UniTBv), 1 Politehnicii Street, 500024 Brasov, Romania; danaluca@unitbv.ro

**Keywords:** infill pattern, infill density, compression uniaxial, additive manufacturing, 3D printed

## Abstract

Additive manufacturing, particularly Fused Deposition Modeling (FDM), has emerged as a promising technology for construction applications due to its ability to fabricate complex geometries, optimize material usage, and enable customized designs. This study investigates the effects of different infill patterns and densities on the compressive behavior of polylactic acid (PLA) specimens to identify suitable configurations for industrial applications. Cubic specimens (50 mm × 50 mm × 50 mm) were designed in SolidWorks, sliced with PrusaSlicer using various conventional and bio-inspired infill patterns, and manufactured using a Prusa i3 MK3S printer. Compression tests were conducted to evaluate stiffness, strength, toughness, and deformation capacity. In addition, finite element method (FEM) simulations were carried out to predict the mechanical response under compressive loading. The results showed that the honeycomb infill pattern provided the best overall performance, combining moderate stiffness with high deformation capacity, reaching strains of approximately 40% before failure, and exhibiting superior energy absorption. Among the tested configurations, an infill density of 20% offered the best balance between mechanical performance, material consumption, and printing time. These findings demonstrate that honeycomb-based structures with moderate infill densities are promising candidates for lightweight engineering applications requiring high energy dissipation and damage tolerance.

## 1. Introduction

Additive Manufacturing (AM), also known as 3D printing, enables the fabrication of three-dimensional objects by depositing material layer by layer from a digital model [1]. Among the available AM technologies, Fused Filament Fabrication (FFF)—also referred to as Fused Deposition Modeling (FDM)—stands out for its low cost, accessibility, and versatility in processing a wide range of thermoplastic materials [2,3,4,5]. In FDM, a filament is melted through a heated nozzle and deposited onto a build platform, building the part layer by layer. Common materials include polylactic acid (PLA), acrylonitrile butadiene styrene (ABS), and glycol-modified polyethylene terephthalate (PETG) [6,7,8]. Among them, PLA is particularly favored due to its renewable origin, biodegradability, and good balance of processability and mechanical performance, making it suitable for functional prototypes, molds, and engineering components [9,10].

The mechanical performance of FFF-printed PLA parts is strongly governed by internal architecture parameters, particularly infill pattern, infill density, layer height, wall thickness, and printing speed, which control load transfer mechanisms and stress distribution within the printed structure [11,12]. While the influence of these parameters on tensile and impact behavior has been extensively studied, their effect on the compressive response is comparatively less explored, despite compression being a critical loading mode in structural, packaging, and energy-absorption applications.

The potential of cellular and multi-cellular architectures to enhance load distribution and energy absorption has been widely demonstrated, particularly in thin-walled and crashworthy systems [13,14,15,16,17,18]. These concepts have also increasingly been explored in additively manufactured thermoplastic structures, where the internal architecture can be tailored through infill-pattern design. Several studies have addressed compressive performance in FDM-printed structures. Kumar et al. [19] demonstrated that specific geometric designs significantly enhance the energy absorption capacity of 3D-printed polymer composites. In a more recent study, biomimetic hexagonal multi-cellular tubes fabricated by FDM were developed to enhance energy absorption and crashworthiness by optimizing cellular geometry [20]. Maurya et al. [4] reported that infill geometries, such as grid and honeycomb, can introduce dimensional deviations as density increases under compressive conditions. However, a systematic comparison of conventional and bio-inspired infill patterns—including honeycomb, grid, triangles, and gyroid—under uniaxial compression, combined with an assessment of manufacturing efficiency, remains insufficiently addressed in the literature.

Finite Element Analysis (FEA) has become a powerful tool for predicting the mechanical response of FDM-printed structures. Software such as ANSYS allows detailed simulation of stress distribution and deformation behavior, enabling the optimization of design parameters before physical fabrication. However, discrepancies between numerical predictions and experimental results may still arise due to the complexity of internal infill architectures and nonlinear failure mechanisms.

Although previous studies have investigated the influence of infill patterns and infill densities on the mechanical behavior of FDM-printed PLA components, a comprehensive assessment that simultaneously considers mechanical performance, finite element validation, and manufacturing efficiency under compressive loading remains limited.

This paper aims to experimentally and numerically evaluate the influence of different infill patterns and infill densities on the compressive behavior of FDM-printed PLA components using a combined experimental and finite element (FEM) approach. This work complements a previous investigation by the authors [21], which characterized the tensile and impact behavior of PLA+ specimens using a full-factorial design integrating ANOVA and machine learning; the present study extends that work by focusing on compressive loading and numerical simulation with ANSYS Mechanical. The main objective of this study is to identify the infill configuration that strikes the best balance among compressive strength, stiffness, energy absorption, deformation capacity, material consumption, and printing time, thereby offering practical design guidelines for lightweight engineering applications subjected to compressive loading.

## 2. Materials and Methods

### 2.1. Materials

This study used PLA-HD filament (1.75 mm diameter) supplied by Winkle (Salamanca, Spain) [22]. This commercial-grade PLA is an amorphous thermoplastic polymer with a glass transition temperature (Tg) of 55–60 °C, a melt flow rate of 8 g/10 min at 210 °C, and a density of 1.24 g/cm^3^. All specimens were manufactured using PLA-HD filament from the same supplier and batch. Different filament colors were used only for visual identification purposes and were not considered an experimental variable. Its reference mechanical properties include a Young’s modulus of 3500 MPa, a tensile strength of 45 MPa, and a Poisson’s ratio of 0.3, which were used as input parameters for the FEM simulations described in Section 2.4. The manufacturer recommends a nozzle temperature of 190–230 °C and a heated bed temperature of 50–70 °C. Before printing, the filament was stored in a dry environment to minimize moisture absorption, as hydrolytic degradation can affect melt viscosity and interlayer bonding in PLA-based systems. This material was selected to maintain continuity with the group’s ongoing research line [21], extending the mechanical characterization of FFF-printed PLA structures into the compressive domain.

### 2.2. Specimen Preparation and Experimental Procedures

Cubic specimens measuring 50 × 50 × 50 mm were designed in SolidWorks^®^ 2025 software (Dassault Systèmes SolidWorks Corporation, Waltham, MA, USA). The models were exported in STL format and processed in PrusaSlicer version 2.9.4 (Prusa Research a.s., Prague, Czech Republic) to define printing parameters for each configuration. The specimens were fabricated from PLA-HD filament, as described in Section 2.1, on a Prusa i3 MK3S printer (Prusa Research a.s., Prague, Czech Republic).

**Figure 1:**
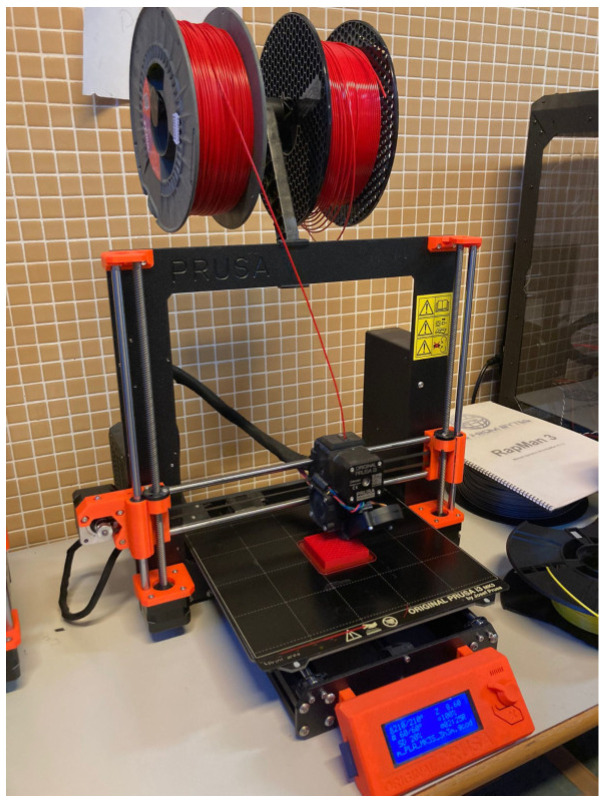
Prusa i3 MK3S printer.

The printing parameters employed in this study are summarized in Table 1. A layer height of 0.30 mm was selected, which is below the nozzle diameter (0.4 mm), ensuring adequate material flow and interlayer adhesion. The printer used for specimen fabrication is shown in Figure 1. All specimens were printed with the 50 mm dimension aligned with the build (Z) direction. During the compression tests, the load was applied parallel to the build direction.

### 2.3. Study of Infill Patterns

**Figure 2:**
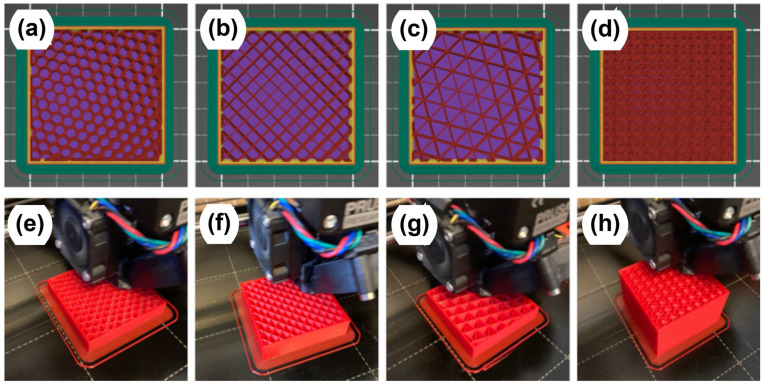
Infill patterns with an infill density of 20%: (**a**,**e**) honeycomb, (**b**,**f**) grid, (**c**,**g**) triangles, and (**d**,**h**) gyroid.

The CAD model was fabricated using four different infill patterns, namely grid, triangles, honeycomb, and gyroid, with an initial infill density of 20%, as illustrated in Figure 2. This density level was selected as the starting point based on findings from a previous study by the authors [21] in which a 20% infill represented a lightweight yet structurally relevant configuration across all evaluated patterns under tensile and impact loading conditions. The objective of this study was to evaluate the influence of infill geometry on the compressive mechanical behavior of printed specimens and to identify the configuration that best balances mechanical performance and manufacturing efficiency. In addition to uniaxial compression tests, printing time and material consumption were considered to assess the suitability of each infill pattern for potential industrial applications. The mechanical performance of the specimens was evaluated in terms of stiffness, compressive strength, and toughness.

The manufacturing specifications estimated by PrusaSlicer and the experimentally measured physical properties of the specimens are summarized in Table 2. Honeycomb (H), Grid (G), Triangles (T), and Gyroid (GY) denote the corresponding configurations evaluated in this study. The experimentally measured physical properties, including measured weight, effective density, and relative density, were determined from five specimens for each configuration, and the reported values represent their average. The measured density was calculated from the average specimen weight and the nominal specimen volume (50 × 50 × 50 mm^3^), while the relative density was obtained by normalizing the effective density with respect to the density of solid PLA (1.24 g/cm^3^). Although all specimens were fabricated with the same nominal infill density (20%), slight differences in measured weight and relative density were observed among the different configurations. These variations are attributed to the distinct internal architectures of each infill pattern, which modify the distribution of deposited material and the effective amount of polymer within the specimens.

### 2.4. Finite Element Method (FEM) Analysis

Before evaluating the effect of infill patterns under uniaxial compression, a finite element method (FEM) analysis was performed for the honeycomb, grid, and triangular geometries using ANSYS Workbench R2 2025 (Ansys Inc., Canonsburg, PA, USA) [23].

The gyroid pattern was excluded from the FEM analysis due to the complexity of its triply periodic minimal surface (TPMS) geometry, which requires significantly finer mesh discretization and substantially higher computational cost to achieve reliable stress field resolution. Its mechanical behavior is therefore characterized exclusively through experimental testing [24].

The CAD models were generated in SolidWorks^®^ software by explicitly modeling the internal infill structures and subsequently imported into ANSYS in STEP format using the Explicit Dynamics module.

The PLA-HD material was defined as an isotropic elastic material with a Young’s modulus of 3500 MPa, a tensile strength of 45 MPa, and a Poisson’s ratio of 0.3 [25], consistent with the values reported in Section 2.1. Given that the objective of the present FEM analysis was to compare stress distributions and load transfer mechanisms among different infill geometries, a linear elastic material model was adopted. Therefore, the simulations were not intended to reproduce the complete nonlinear compressive response, including mechanisms such as yielding, fracture, buckling, or progressive collapse.

**Figure 3:**
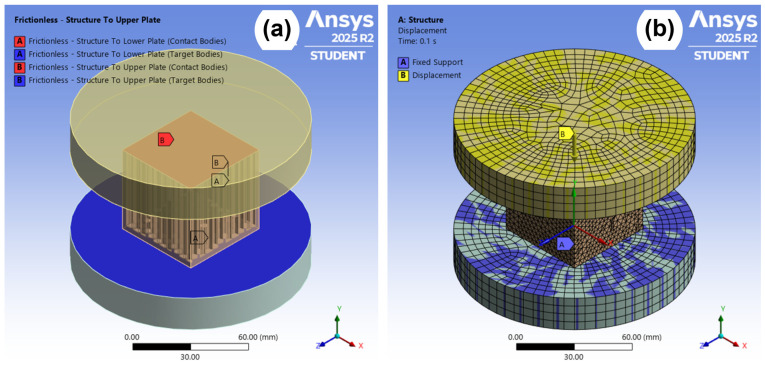
Finite element model: (**a**) frictionless contact regions between the structure and the lower and upper plates (target bodies (blue) and contact bodies (red)); and (**b**) boundary conditions (fixed support (purple) and imposed downward displacement (yellow)) and the finite element mesh with an average element size of 3 mm.

The uniaxial compression setup was represented using two rigid steel plates (Ø 125 mm, 20 mm thick) to reproduce the experimental boundary conditions. The lower plate was fully fixed, constraining all degrees of freedom (translations and rotations), while the upper plate was modeled as a rigid body subjected to an imposed displacement [26] of 2.5 mm, corresponding to an axial strain of approximately 5% relative to the specimen height of 50 mm. Frictionless contact conditions were assumed between the specimen and both plates to minimize external friction effects and focus on the intrinsic load transfer behavior and stress distribution of the infill architectures. Since the FEM analysis was intended for comparative evaluation rather than exact failure prediction, frictionless contact was considered an appropriate simplification. The frictionless contact regions between the structure and the loading plates are shown in Figure 3a, while the boundary conditions and finite element mesh are shown in Figure 3b.

The finite element discretization was performed using linear elements with an average element size of 3 mm, and the global mesh resolution was set to 4, as shown in Figure 3b. The resulting meshes comprised 60,856 elements for the honeycomb structure, 101,150 elements for the grid structure, and 62,162 elements for the triangular geometry. Mesh quality was assessed using the element quality metric, with average values of 0.58, 0.45, and 0.49 for the honeycomb, grid, and triangular structures, respectively, all within acceptable limits, confirming numerical stability. Due to the computational limitations of the student version of ANSYS, a formal mesh convergence analysis was not performed. However, the selected mesh density was considered adequate for the objectives of this study, capturing the main stress distribution features and load transfer mechanisms of the different infill geometries.

The analysis was carried out using an explicit formulation under quasi-static conditions, with a total simulation time of 0.1 s to ensure progressive load application and minimize dynamic inertial effects.

The validity of the quasi-static assumption was assessed using an energy-based criterion reported in the literature [27] and consistent with the recommendations provided in ANSYS Explicit Dynamics guidelines [28]. According to this criterion, the response is considered quasi-static when the ratio of kinetic energy to internal energy remains below 5% throughout the deformation process.

### 2.5. Mechanical Characterization

**Figure 4:**
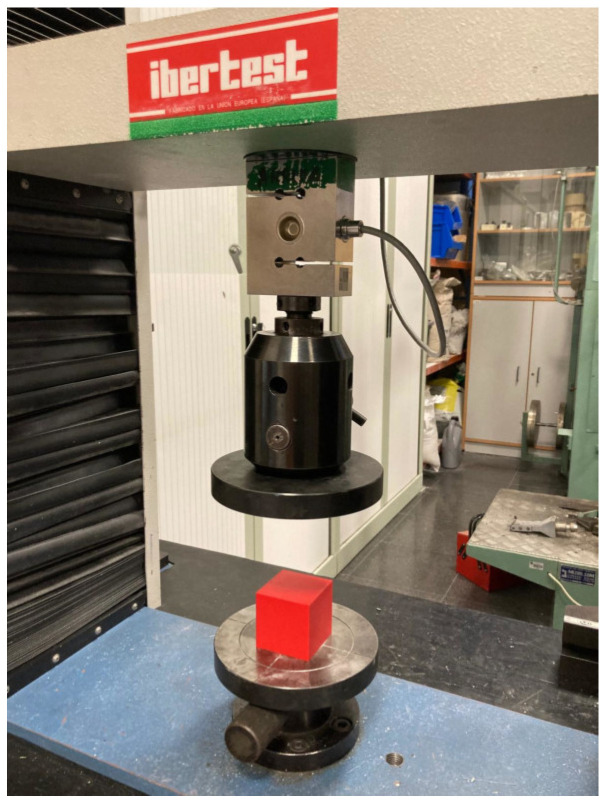
Electromechanical universal testing machine (ELIB 30) and uniaxial compression test of the structure.

The mechanical characterization of the 3D-printed specimens was carried out using compression tests performed on an ELIB 30 universal electromechanical testing machine (Ibertest, Madrid, Spain) equipped with a 50 kN load cell (Figure 4). All tests were conducted in accordance with ISO 604:2002 [29] at a crosshead speed of 5 mm/min. For each infill configuration, five specimens were tested, and the reported mechanical parameters correspond to the average values obtained from these replicates. All experiments were performed at room temperature, and the stress–strain data were recorded during the compression tests.

The mechanical parameters were determined from the experimental stress–strain curves. The yield stress (σ_y_) was defined as the stress value corresponding to the transition from the elastic to the plastic deformation region. The compressive modulus (E_c_) was calculated from the slope of the initial linear elastic region of the stress–strain curve. The resilience (E_R_) was obtained from the area under the curve up to the yield point, representing the elastic energy absorbed by the specimen. The toughness (E_T_) was calculated as the area under the stress–strain curve up to failure, representing the total energy absorption capacity. The strain at break (εB) was defined as the strain measured at the specimen’s failure point. These parameters were used to evaluate the influence of infill architecture on the compressive mechanical response of the printed structures.

## 3. Results and Discussion

### 3.1. FEM-Based Simulation and Experimental Data on the Influence of Infill Patterns

**Figure 5:**
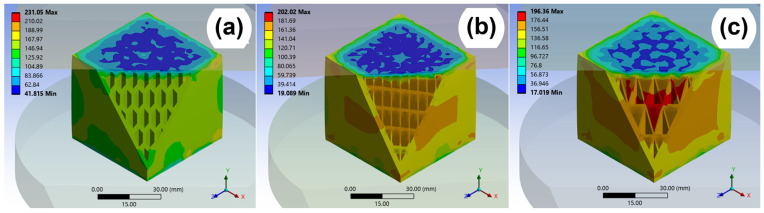
FEM-simulated sectional stress contours for (**a**) honeycomb, (**b**) grid and (**c**) triangles infill structures under compressive loading.

In this study, sectional stress-contour images from FEM simulations with a prescribed displacement of 2.5 mm are presented to identify regions with the highest stress concentrations. These cut-plane representations provide insight into the internal stress distribution within each infill structure and highlight the critical areas where failure is likely to initiate, as shown in Figure 5.

The FEM results indicate that the honeycomb structure reaches a maximum von Mises stress of 231 MPa, exceeding the values obtained for the grid and triangular patterns (202 MPa and 196 MPa, respectively). Since the model assumes linear elastic behavior, the obtained stress values should be interpreted as indicators of relative stress concentration rather than absolute failure stresses. Regions with higher stress levels are considered potential critical locations where damage may initiate; however, the exact failure mechanisms cannot be predicted using the present linear elastic model. The honeycomb pattern exhibits a more homogeneous stress distribution with intermediate stress levels of around 147 MPa, suggesting efficient load redistribution. In contrast, the grid pattern shows pronounced stress concentrations at nodal and corner regions (up to 161 MPa), while the triangular pattern develops localized high-stress regions along primary load paths, both indicating preferential sites for damage initiation.

The numerical results reveal clear differences in stress distribution among the evaluated geometries.

The energy balance of the simulations confirmed the validity of the quasi-static assumption (Table 3). The Ec/Ei ratio remained below 0.01% in all cases, confirming that inertial effects are negligible [27]. Among the evaluated configurations, the honeycomb structure exhibited the highest elastic internal energy (232.61 J), followed by the grid (202.26 J) and triangular (191.32 J) patterns. These values should be interpreted as comparative indicators of elastic load transfer rather than direct predictions of experimental energy absorption capacity, which is evaluated from the compression tests.

**Figure 6:**
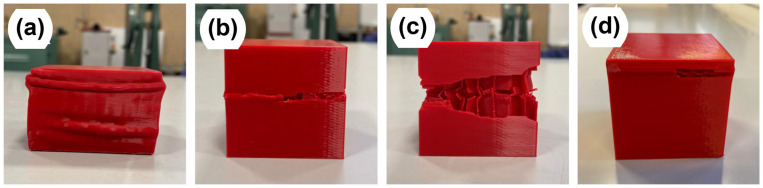
Images of specimens under compressive loading up to fracture: (**a**) honeycomb, (**b**) grid, (**c**) triangles, and (**d**) gyroid.

**Figure 7:**
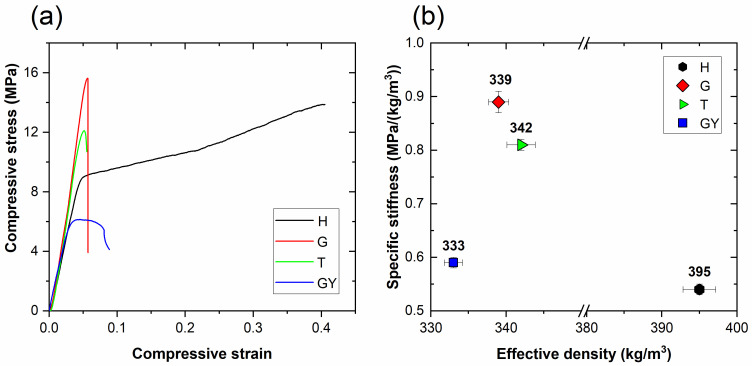
(**a**) Compressive stress–strain curves from experimental testing of 3D-printed infill patterns; (**b**) specific stiffness (effective modulus/effective density) as a function of effective density for each infill pattern.

The experimentally tested specimens are shown in Figure 6, and the corresponding stress–strain curves are presented in Figure 7a. The mechanical parameters are summarized in Table 4. All reported values correspond to the average of five specimens for each configuration. The deformation pattern of cellular structures is closely related to their energy absorption capacity, as it reflects the underlying mechanisms governing compressive deformation and energy dissipation [30]. The honeycomb structure exhibits the highest deformation capacity, reaching strains close to 40% before failure, with a compressive strength of 8.52 MPa and a toughness of approximately 3998 kJ/m^3^. The grid and triangular patterns reach higher compressive strengths (15.4 MPa and 11.7 MPa, respectively) but fail at strains below 10%. In comparison, the gyroid exhibits the lowest compressive strength (5.78 MPa) alongside the highest elastic modulus (302 MPa).

The stress–strain curves in Figure 7a show an initial linear elastic region followed by progressive damage accumulation. Cellular materials typically exhibit three deformation stages: (i) initial elastic deformation, (ii) a post-yield plateau associated with progressive collapse mechanisms, and (iii) densification. In the present study, only the initial elastic regime is fully developed in the present specimens, likely due to their relatively compact internal architecture. This is consistent with Mohotti et al. [31], who reported that multi-stage collapse is primarily observed in highly porous lattice structures.

Regarding energy absorption, the grid pattern shows the highest resilience (393 kJ/m^3^), while the honeycomb structure shows the highest toughness (3998 kJ/m^3^). As shown in Figure 7b, the grid and triangular configurations achieve the highest specific stiffness (≈0.89 and ≈0.80 MPa·m^3^/kg), whereas honeycomb and gyroid show lower values (≈0.54 and ≈0.59 MPa·m^3^/kg, respectively), despite the honeycomb pattern having the highest effective density (~395 kg/m^3^). The superior toughness and deformation capacity of the honeycomb structure are consistent with previous studies reporting enhanced energy absorption in 3D-printed multicellular architectures [20]. Furthermore, previous studies have demonstrated that variations in cellular and lattice geometry significantly influence the balance between compressive stiffness, load-bearing capacity, deformation, and energy absorption [32,33]. This is consistent with the present findings, where changes in infill architecture resulted in distinct combinations of compressive strength, stiffness, deformation capacity, and energy absorption.

Overall, both FEM and experimental results consistently identify the honeycomb pattern as having superior energy and deformation capacities due to its efficient load redistribution mechanism. In contrast, the grid and triangular configurations show higher stiffness and strength but reduced ductility, leading to premature failure. The gyroid structure presents intermediate behavior with lower strength but relatively high stiffness. Therefore, the most suitable infill configuration depends on the intended application. Honeycomb is preferable for applications requiring energy absorption, deformation capability, and damage tolerance, whereas the grid pattern is more appropriate for load-bearing applications where higher compressive strength, stiffness, and elastic energy storage are prioritized. This agrees with Al-Kharusi and Al Owiemri, who reported superior compressive performance of honeycomb due to more efficient stress distribution and load transfer [34]. Furthermore, the strong dependence of mechanical behavior on infill geometry, which governs deformation and failure mechanisms, agrees with the findings reported by Aboelella et al. [35].

### 3.2. Study of Infill Density

**Figure 8:**
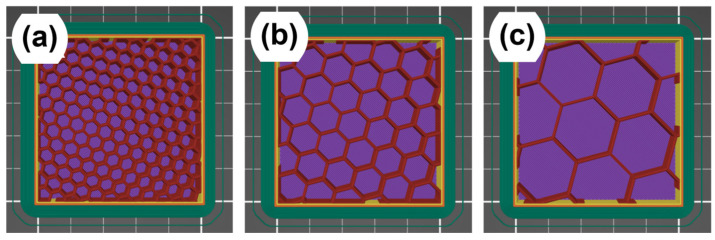
Infill density of honeycomb: (**a**) H20, (**b**) H10 and (**c**) H5.

Based on the results obtained in Section 3.1, the honeycomb pattern was selected for the infill density study. The selected infill densities (5%, 10%, and 20%) were chosen to represent different levels of material utilization, ranging from highly lightweight configurations to a more mechanically robust condition. The 20% infill density was selected as the reference condition because it provides a balance between mechanical performance and material efficiency. Lower infill densities (10% and 5%) were included to evaluate the effect of material reduction on compressive behavior, energy absorption, and manufacturing efficiency. These configurations are shown in Figure 8, while the corresponding manufacturing characteristics estimated by PrusaSlicer and the experimentally measured physical properties of the honeycomb specimens, including measured weight, effective density, and relative density, are summarized in Table 5.

#### Influence of Infill Density

**Figure 9:**
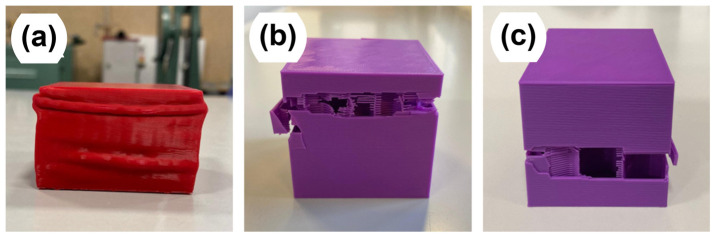
Images of honeycomb specimens with different infill densities under compressive loading up to fracture: (**a**) H20, (**b**) H10, and (**c**) H5. Different filament colors were used only for visual identification purposes and were not considered an experimental variable.

**Figure 10:**
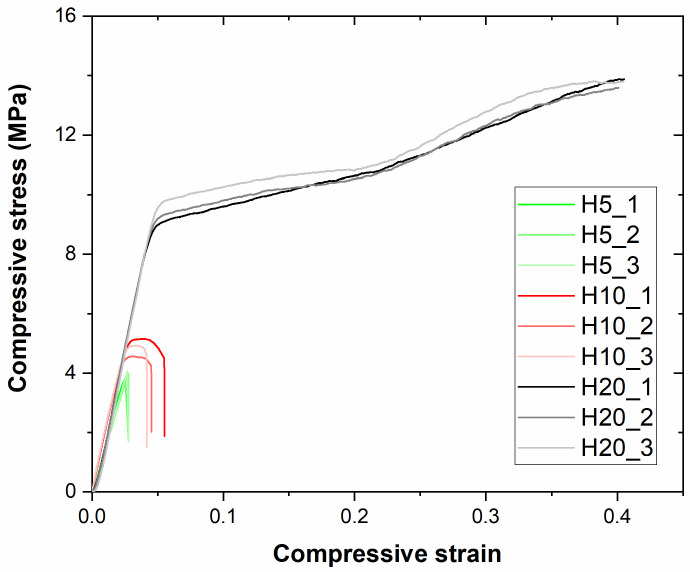
Compressive stress–strain curves of three specimens for each honeycomb infill density (5%, 10%, and 20%).

Figure 9 shows the honeycomb specimens with different infill densities (5%, 10%, and 20%) after compressive testing up to fracture, and Figure 10 presents the stress–strain curves obtained for the three specimens tested at each infill density. The results confirm a strong dependence of compressive behavior on infill density. The 20% configuration achieves the highest compressive strength (≈8.5 MPa) and the greatest deformation capacity (≈40% strain before failure). At lower densities, compressive strength drops to approximately 4.8 MPa at 10% and 4.0 MPa at 5%, accompanied by a reduction in strain-to-failure, indicating a progressive loss of structural ductility. The post-compression images also reveal localized collapse and damage within the honeycomb architecture, particularly in the H10 and H5 specimens, where openings and disrupted internal regions are evident (Figure 9).

This behavior can be attributed to a reduction in structural continuity within the internal honeycomb network as density decreases, thereby limiting load redistribution and the overall capacity to sustain compressive loading before failure. This finding is in agreement with previous studies reporting that higher infill percentages enhance internal connectivity and mechanical performance [34].

From a manufacturing perspective, reducing infill density yields significant gains in fabrication efficiency: printing time decreases from 210 min at 20% to 106 min at 10% and 77 min at 5%, with proportional reductions in material consumption. These trends are consistent with the experimentally measured properties reported in Table 5. The measured specimen weight decreases from 49.36 g for H20 to 34.18 g for H10 and 26.60 g for H5. Similarly, the effective density decreases from 395 kg/m^3^ to 273 kg/m^3^ and 213 kg/m^3^, while the relative density decreases from 0.319 to 0.220 and 0.172, respectively. Although the estimated and experimentally measured values differ slightly, both confirm a progressive reduction in material content as infill density decreases. However, this reduction in material content is accompanied by a substantial decrease in mechanical performance. Therefore, the 20% configuration represents the best balance among the tested densities, combining adequate compressive strength, high energy absorption, and reasonable manufacturing efficiency.

## 4. Conclusions

This study investigated the influence of infill pattern and infill density on the compressive mechanical behavior of FDM-printed PLA structures using a combined experimental and finite element (FEM) approach. The internal architecture strongly influenced the compressive response, with the honeycomb pattern exhibiting the highest deformation capacity and toughness, reaching approximately 40% strain before failure and a toughness of nearly 4000 kJ/m^3^. FEM analysis showed a more uniform stress distribution in the honeycomb structure compared to the grid and triangular geometries, providing mechanistic insight into the experimental observations that would not be accessible through testing alone. In contrast, the grid pattern achieved the highest stiffness and resilience (specific stiffness ≈ 0.89 MPa·m^3^/kg; resilience = 393 kJ/m^3^), while the triangular and gyroid structures showed intermediate and lower performance, respectively. Regarding infill density, a trade-off was identified between mechanical performance and manufacturing efficiency. Although reducing the density from 20% to 5% decreased printing time by ~63% and material consumption proportionally, it led to a substantial reduction in compressive strength and ductility. The 20% honeycomb configuration provided the best balance among the tested conditions.

Overall, honeycomb structures are more suitable for applications requiring energy absorption, deformation capability, and damage tolerance, whereas grid structures are preferable for lightweight structural applications requiring higher stiffness and compressive strength. These findings confirm that FEM simulation, combined with experimental validation, enables both the rationalization of failure mechanisms and the optimization of infill design. Future work should incorporate nonlinear material behavior and progressive damage criteria into the FEM model, as well as extend this characterization to a broader density range and dynamic loading conditions.

## Figures and Tables

**Table 1:** Printing parameters used for specimen fabrication.

Property	Value
Nozzle Diameter (mm)	0.4
Layer Height (mm)	0.3
Extruder Temperature (°C)	215
Bed Temperature (°C)	60
Print Speed (mm/s)	50
Number of perimeters	2
Wall Thickness (mm)	1.14
Top/Bottom Layers	4/4
Extrusion Width (mm)	0.5
Raster Angle (°)	45
Base Layer Type	Brim

**Table 2:** Manufacturing specifications estimated by PrusaSlicer and experimentally measured physical properties for specimens printed with 20% infill density.

Parameters	H	G	T	GY
Time of print (min)	210	95	105	138
Weight of print (g)	54.05	46.95	47.05	45.25
Material length used (m)	15.78	15.74	15.78	15.17
Measured weight (g)	49.36 ± 0.27	42.34 ± 0.17	42.70 ± 0.23	41.64 ± 0.15
Effective density (kg/m^3^)	395	339	342	333
Relative density	0.319	0.273	0.276	0.269

**Table 3:** Energies and quasi-static verification.

Pattern	Ec (J)	Ei (J)	Ec/Ei (%)
Honeycomb	0.00793	232.61	0.0034%
Grid	0.00673	202.26	0.0033%
Triangles	0.00640	191.32	0.00335%

**Table 4:** Mechanical properties of PLA-HD infill patterns.

Code	σ_y_ (MPa)	σM (MPa)	σB (MPa)	ε_y_	εM	E_c_ (MPa)	E_R_ (kJ/m^3^)	E_T_ (kJ/m^3^)
H	8.52 ± 1.10	13.4 ± 0.81	-	0.044 ± 0.006	0.398 ± 0.003	212 ± 3.40	212 ± 3.40	3998 ± 298
G	15.4 ± 0.11	15.5 ± 0.08	4.60 ± 0.56	0.054 ± 0.002	0.056 ± 0.002	302 ± 6.08	393 ± 9.57	448 ± 23.86
T	11.7 ± 0.26	11.7 ± 0.24	10.33 ± 1.05	0.049 ± 0.006	0.051 ± 0.001	275 ± 2.16	189 ± 40.75	315 ± 32.75
GY	5.78 ± 0.76	6.14 ± 0.79	5.31 ± 0.74	0.031 ± 0.004	0.046 ± 0.003	197 ± 2.70	102 ± 24.21	362 ± 107.30

**Table 5:** Printing characteristics estimated by PrusaSlicer and experimentally measured physical properties of honeycomb specimens manufactured with different infill densities.

Parameters	H20	H10	H5
Time of print (min)	210	106	77
Weight of print (g)	54.05	37.08	27.55
Material length used (m)	15.78	12.43	9.24
Measured weight (g)	49.36 ± 0.27	34.18 ± 0.22	26.6 ± 0.00
Effective density (kg/m^3^)	395	273	213
Relative density	0.319	0.220	0.172

## Data Availability

The original contributions presented in this study are included in the article. Further inquiries can be directed to the corresponding author.
